# Adipose triglyceride lipase promotes the proliferation of colorectal cancer cells via enhancing the lipolytic pathway

**DOI:** 10.1111/jcmm.16349

**Published:** 2021-02-23

**Authors:** Haofan Yin, Wentao Li, Laiming Mo, Shaotuan Deng, Weijia Lin, Caiqi Ma, Zhaofan Luo, Chuanghua Luo, Honghai Hong

**Affiliations:** ^1^ Department of Clinical Laboratory The Seventh Affiliated Hospital of Sun Yat‐sen University Shenzhen China; ^2^ Reproductive Medical Center Guangzhou Women and Children's Medical Center of Sun Yat‐sen University Guangzhou China; ^3^ State Key Laboratory of Oncology in South China Collaborative Innovation Center for Cancer Medicine Sun Yat‐sen University Cancer Center Guangzhou China; ^4^ Department of Clinical Laboratory The Third Affiliated Hospital of Guangzhou Medical University Guangzhou China

**Keywords:** adipose triglyceride lipase, colorectal cancer, lipolytic, proliferation

## Abstract

Abnormal lipid metabolism is the sign of tumour cells. Previous researches have revealed that the lipolytic pathway may contribute to the progression of colorectal cancer (CRC). However, adipose triglyceride lipase (ATGL) role in CRC cells remains unclear. Here, we find that elevated ATGL positively correlates with CRC clinical stages and negatively associates with overall survival. Overexpression of ATGL significantly promotes CRC cell proliferation, while knockdown of ATGL inhibits the proliferation and promotes the apoptosis of CRC cells in vitro. Moreover, in vivo experiments, ATGL promotes the growth of CRC cells. Mechanistically, ATGL enhances the carcinogenic function of CRC cells via promoting sphingolipid metabolism and CoA biosynthesis pathway‐related gene levels by degrading triglycerides, which provides adequate nutrition for the progression of CRC. Our researches clarify for the first time that ATGL is a novel oncogene in CRC and may provide an important prognostic factor and therapeutic target for CRC.

## 1 INTRODUCTION

Colorectal cancer (CRC) is the fourth most common cancer worldwide and the fifth leading cause of cancer death in humans.^1^ At present, people's eating habits are mainly high‐fat diet and lack of high dietary fibre intake, which makes the incidence rate of CRC showing a rising trend.^1^ The characteristics of the CRC progression are rapid infiltrating growth, early metastasis and unfavourable prognosis.^2^

Unlike most normal cells, cancer cells exhibit uncontrolled cell proliferation. To cope with the unlimited growth, expansion and diffusion, cancer cells must efficiently generate energy, even in the microenvironment of hypoxia and lack of nutrition.^3^ Warburg effect shows that cancer cells have increased glucose uptake and glycolysis dependent metabolism.^4^ Remarkably, the lipolytic pathway is also reprogrammed in many types of cancer cells, which are depend on mitochondrial β‐oxidation.^5^ CRC cells have been in the intestinal high‐fat environment for a long time, which leads to the active function of β‐oxidation. Therefore, it is worthy to explore the lipolytic enzyme function in CRC.

At present, the main reported enzymes involved in lipolysis are adipose triglyceride lipase (ATGL), hormone‐sensitive lipase (HSL) and monoacylglycerol lipase (MAGL).^6^ Among them, ATGL is the most critical rate‐limiting enzyme, which is a member of the patatin‐like phospholipase domain (PNPLA) family.^7^ Also, the distribution of ATGL in normal human was mainly in adipose tissue, while medium to low expression was detected in other tissues.^7^, ^8^ Triglyceride (TAG) metabolism is initiated by ATGL through hydrolysing TAG into free fat acid (FFA) and diacylglycerol (DAG).^9^ The next step is to decompose DAG into monoacylglycerol (MAG), which requires HSL. Lastly, MAG is further broken down by MAGL into FFA and glycerol.^10^ In addition to energetic purposes, FFA is essential for membranes biosynthesis and also serves as a signalling molecule.^11^ ATGL‐mediated lipolysis releases a large amount of FFA, which is important to adapt to the high proliferation rates of tumour cells. Previous studies have shown that the lipolytic enzyme, MAGL, enhances tumour growth and metastasis via the FFA pathway.^12^, ^13^ But recent researches have also suggested that MAGL has an antitumour effect.^14^ Lipolytic enzymes roles need to be further explores in tumour development. ATGL have been shown to promote the proliferation of hepatocellular carcinoma cells.^15^, ^16^ However, it is not clear whether ATGL promotes tumour growth or other functions in CRC.

Our study investigated the role of ATGL, a key new oncogene in the development of CRC. And we determined the hypothesis that ATGL promoted CRC proliferation by enhancing lipolysis. These collective findings of our research might provide a novel target for the treatment of CRC.

## 2 MATERIALS AND METHODS

### 2.1 Tumour xenograft

We bought 4‐week‐old male BALB/c nude mice from Beijing Vital River Laboratory Animal Technology Co., Ltd. The mice were randomly divided into 2 groups based on a previously described standard protocol. 1 × 10^6^ SW480‐Vector or SW480‐ATGL cells were injected into the inguinal folds of mice, respectively (n = 6 in each group). And a nude mouse injected with SW480‐Vector cells did not form tumour. We measured the tumour volume with an external calliper and calculated its results by the following formula: Volume = length × width^2^⁄2. The mice were killed at 27 days after injection. Then, tumours were dissected, weighed, photographed and stored at −80°C for further researches.

### 2.2 Human samples

The CRC tissue microarrays (HColA180Su21‐1‐250, 94 cases) were purchased from Shanghai Outdo Biotech. All procedures were performed under consensus agreements and in accordance with the Chinese Ethical Review Committee. The clinical and biological characteristics of the patients were described in Table 1.

**TABLE 1:** Correlation between expression of ATGL and clinicopathological features in 94 cases of CRC

Characteristics	No. of patients	Expression of ATGL		*P* value
		Low	High	
Patients				
Malignant tumour	94	45	49	<0.001
Adjacent tumour	86	71	15	
Age				
≤64	42	18	24	0.382
>64	52	27	25	
Gender				
Female	48	26	22	0.212
Male	46	19	27	
Clinical stage				
I+II	64	36	28	0.018
III+IV	30	9	21	
T classification				
T1‐T2	9	8	1	0.010
T3‐T4	85	37	48	
N classification				
N0‐N1	77	40	37	0.092
N2‐N3	17	5	12	
M classification				
M0	89	43	46	0.717
M1	5	2	3	
Intravascular tumour thrombus				
Negative	46	33	13	0.247
Positive	48	29	19	
Nerve invasion				
Negative	76	38	38	0.396
Positive	18	7	11	
CD8 expression				
Low	63	24	39	0.007
High	31	21	10	
PDL1 expression				
Low	79	36	43	0.305
High	15	9	6	
PD1 expression				
Low	82	47	35	0.947
High	12	7	5	

### 2.3 Cell lines and culture

The human CRC cell lines (HCI‐H508, SW480, CaCO2, LoVo, HCT116, SW620, HT29) were obtained from the American Type Culture Collection. The normal intestinal epithelial cell lines (CCD841) were provided by Professor Peng Huang, from Sun Yat‐sen University Cancer Center. Cell lines were authenticated by Cellcook Biotech Co., Ltd.

### 2.4 Western Blotting

The total proteins were collected using SDS lysis buffer (Beyotime, P0013G), and protein concentration was determined by Bicinchoninic Acid (KeyGen, KGP902). The following primary antibodies were used: ATGL (ab85348) from Abcam; β‐actin (A5441) from Sigma‐Aldrich. HRP‐conjugated anti‐rabbit IgG (Cell Signaling Tech, #7074) and anti‐mouse IgG (Sigma‐Aldrich, AP308P) were used as secondary antibodies. Proteins were determined using ECL Plus Reagent (Millipore, WBKLS0100).

### 2.5 RNA isolation and RT‐qPCR

Total RNA was extracted using TRIzol (Thermo Scientific, 15596026) according to the manufacturer's protocol. First‐strand cDNA synthesis was performed using 500 nanograms of total RNA, and the RT‐qPCR analysis system was performed using iQ SYBR Green Supermix and the iCycler Real‐time PCR Detection System (Bio‐Rad).

### 2.6 Immunofluorescence staining

After fixed in 4% paraformaldehyde, cells were blocked with goat serum at 37°C for 1hour. They were incubated with rabbit Ki67(Millipore, AB9260) antibodies at 4°C overnight, then were incubated with FITC conjugated goat anti‐rabbit IgG (Dako, K500711) at 37°C for 1 hours after three times washing. Finally, the cell nucleus was stained with DAPI (Sigma‐Aldrich, D9542).

### 2.7 Cell Counting Kit‐8 (CCK8)

Cell proliferation was measured via cell viability with a Cell Counting Kit‐8 (Dojindo). CRC cells were seeded into 96‐well plates and cultured for 24, 48 and 72 hours. Then, 10 μL CCK8 reagent was added to 96‐well plates and incubated for 2 hours. The absorbance (OD450 nm) was measured using a microplate reader (TECAN) and calculated.

### 2.8 Colony formation assay

CRC cells were plated in 6‐well dishes (500 cell/dish) and then incubated for 2 weeks for colony formation. After 14 days, cell colonies were then fixed in 4% polyformaldehyde and stained with 0.1% crystal violet. All colonies were counted separately for each sample, and the relative colony numbers were calculated.

### 2.9 Terminal deoxynucleotidyl transferase‐mediated dUTP nick end labelling (TUNEL) staining

Tissue sections were deparaffinized and hydrated in xylene and gradient concentrations of ethanol, then incubated in proteinase K at room temperature for 30 minutes and stained with TUNEL kit (Sigma‐Aldrich). Label solution was used instead of TUNEL reagent in the negative control group. All the images were captured by a fluorescence microscope (DFC700T, Leica). Cells that were positive for TUNEL staining and aligned with DAPI staining were considered apoptotic cells and counted.

### 2.10 siRNA and lentivirus

ATGL siRNA and control siRNA were purchased from RiboBio. According to the manufacturer's instructions, transfections were performed at approximately 60% confluency using RNAiMAX (Invitrogen). After 48 hours, confirmation of interference was carried out using real‐time quantitative PCR (RT‐qPCR) and Western blotting. Plasmids encoding ATGL were packaged as lentivirus by GeneChem Co., Ltd.

### 2.11 Annexin V/propidium iodide flow cytometric analysis

CRC cell staining with Annexin V and PI was carried out using an Annexin V‐FITC/PI Apoptosis Detection kit (Merck). A total of 1 × 10^6^ cells were incubated at 37°C for 30 minutes before centrifugation to collect the cell pellet, then resuspended in a Ca2+‐enriched binding buffer and analysed using a Beckman Coulter flow cytometry. Data were calculated using Cell Quest software.

### 2.12 Measurement of free fatty acids

Different groups of SW480 and HCT116 cells were treated with either BSA or 400 μM oleic acid (OA) for 6 hours, then washed and incubated with phenol red‐free DMEM complexed with 1% BSA for additional 24 hours. The supernatant was collected and subjected to subsequent analyses. FFA from cell culture medium was measured using the EnzyChromTM Free Fatty Acid Assay Kit, according to the manufacturer's instructions. FFA was calculated from a standard curve for each assay, and the data were normalized to total protein.

### 2.13 Oil Red O staining

For lipid droplet staining, different groups of SW480 and HCT116 cells incubated with 400 μM OA for 6 hours, or 10 μm cryostat sections from indicated CRC xenografts, were washed, fixed in 4% paraformaldehyde for 10 minutes and rinsed with 60% isopropanol. The slides were then placed in the freshly prepared working Oil Red O solution for 10 minutes at room temperature and rinsed again with 60% isopropanol. After lightly stained nuclei with haematoxylin and washed with distilled water, the slides were covered with glycerine jelly that will harden after a few hours. Relative lipid content was quantified by using Image Pro Plus 6.0.

### 2.14 TCGA data analysis

The RNASeq data and clinical data for CRC were obtained from The Cancer Genome Atlas (TCGA) databases (https://genome‐cancer.ucsc.edu). For the association of ATGL expression with survival was used as a surrogate end‐point and patients dichotomized by ATGL expression. Gene set enrichment analysis (GSEA) was used based on CRC TCGA databases.

### 2.15 Statistical analysis

We presented the variability of the data as the SD (mean ± SD). Between two groups, significant differences were determined using Student's *t* test. And we used one‐way ANOVA to assessed multiple groups significant differences. χ^2^ test was used to determine the relationships between clinicopathological characteristics and ATGL expression. Survival curves were plotted by the Kaplan‐Meier method and further compared by the log‐rank test. Statistical significance was defined at *P* < 0.05.

## 3 RESULTS

### 3.1 Elevated ATGL is associated with tumour progression in CRC

**FIGURE 1:**
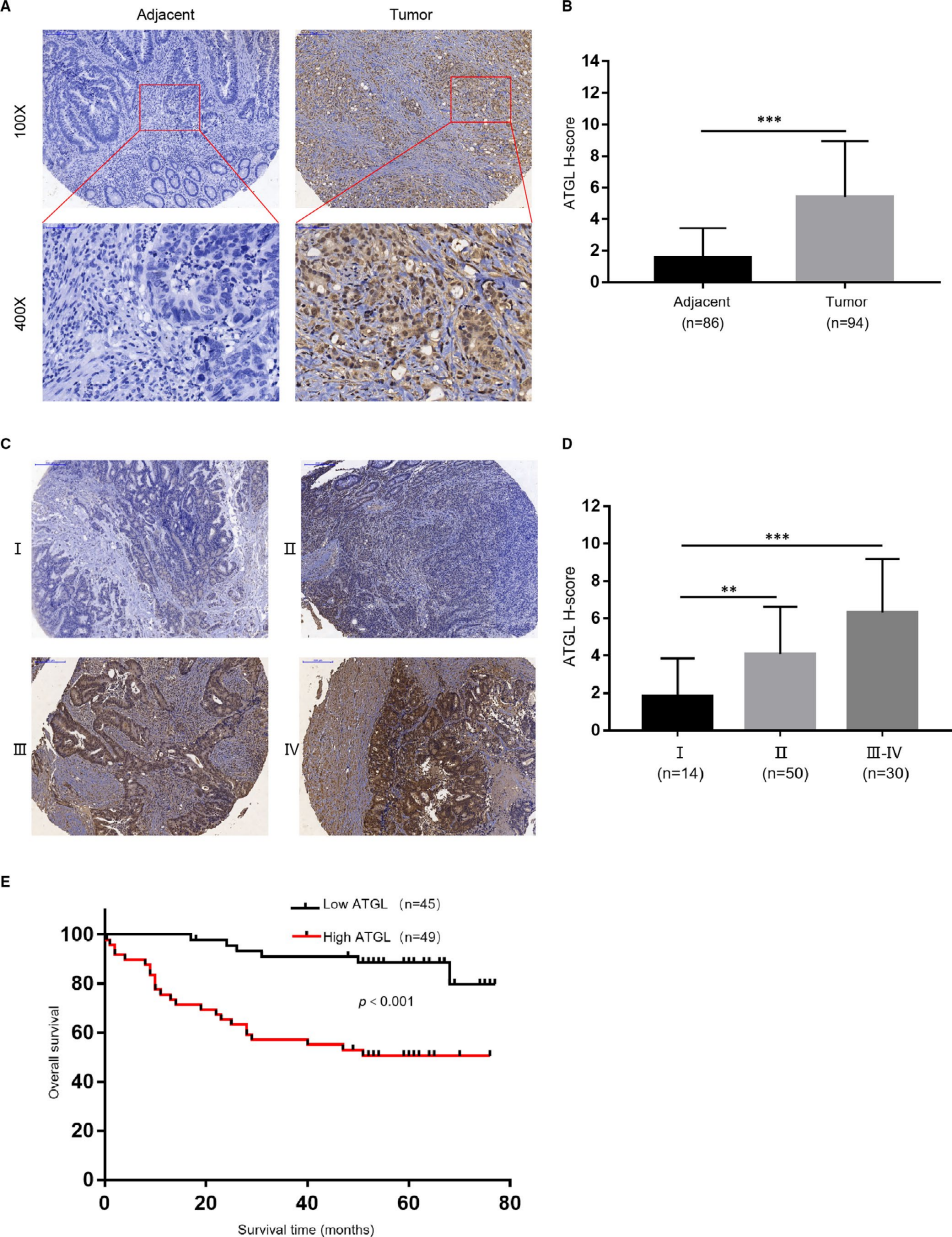
Elevated ATGL is associated with tumour progression in CRC. (A) ATGL IHC staining of 86 adjacent specimens and 94 CRC specimens in CRC Tissue Microarray (top, 100× magnification, Scale bar 200 μm; bottom, 400× magnification, Scale bar 50 μm). (B) Statistical analysis of ATGL staining of adjacent specimens versus CRC specimens. IHC was quantified by staining intensity (SI). H‐Score ≥5 was considered high ATGL expression; ***P < 0.001. (C) Representative images of ATGL IHC staining at different clinical stages (100× magnification). Scale bar 200 μm. (D) Statistical analysis of ATGL staining at different clinical stages; **P < 0.01 and ***P < 0.001 was compared with clinical stage I. (E) Overall survival of CRC patients in CRC Tissue Microarray with low and high ATGL expression

To investigate the underlying role of ATGL in CRC, the expression of ATGL in a CRC Tissue Microarray was detected by immunohistochemistry (IHC) staining. Impressively, ATGL expression was significantly elevated in CRC specimens compared with adjacent specimens (Figure 1A,B). In addition, ATGL expression increased along with the progression of CRC clinical stages (Figure 1C,D). Moreover, the chi‐square test was used to analyse the association between clinicopathological characteristics and ATGL expression. The result indicated that the expression of ATGL was related to the clinical stage (*P* = 0.018), T classification (*P* = 0.010) and CD8 expression (*P* = 0.007), but not with gender, age, N classification, M classification, intravascular tumour thrombus, nerve invasion, PDL1 expression and PD1 expression (Table 1). Meanwhile, Kaplan‐Meier analysis showed that ATGL protein levels were negatively correlated with overall survival (*P* < 0.001; Figure 1E). Collectively, these results suggested that elevated ATGL contributes to the progression of CRC and may be a poor prognostic factor for CRC.

### 3.2 Detection of ATGL expression in CRC cell lines

**FIGURE 2:**
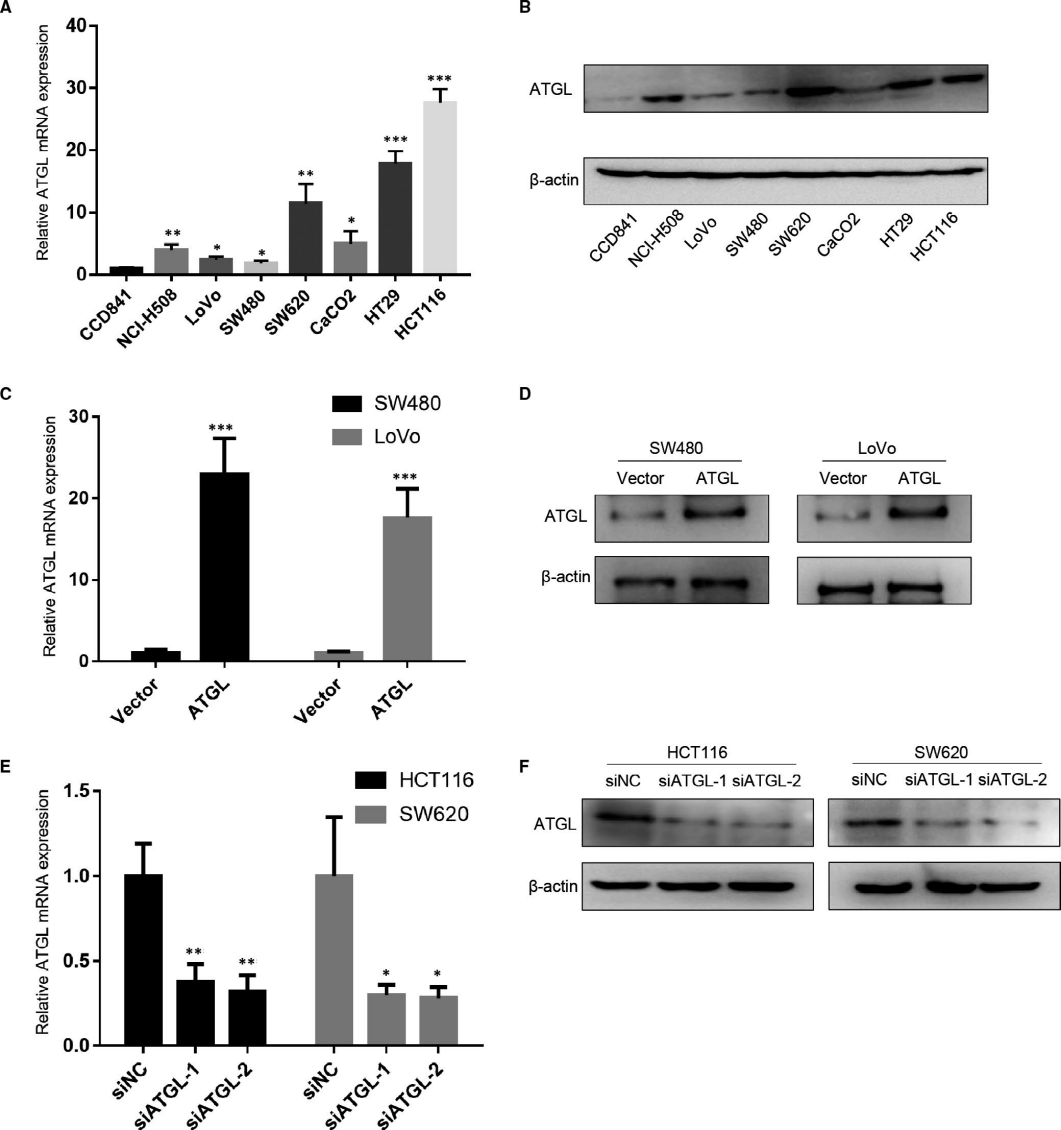
Detection of ATGL expression in CRC cell lines. (A, B) qRT‐PCR analysis (A) and Western blot analysis (B) of ATGL expression in CRC cell lines, and the CCD841 cells were used as normal control. (C, D) Overexpression efficiency of ATGL at mRNA level (C) and protein level (D) in SW480 and LoVo cell lines. (E, F) Knockdown efficiency of ATGL at mRNA level (E) and protein level (F) in HCT116 and SW620 cell lines. Data were represented as the mean ± SD of three independent experiments; *P < 0.05, **P < 0.01, ***P < 0.001

The expression of ATGL in seven CRC cell lines (NCI‐H508, LoVo, SW480, SW620, CaCO2, HT29, HCT116) was presented in the Figure 2A,B, and the normal control was CCD841 cell lines. Both ATGL mRNA and protein expression in the CRC cell lines were remarkably higher than in the immortalized CCD841 human intestinal epithelial cell line (Figure 2A,B). These results also indicated that the expression levels of HCT116 and SW620 cells were higher, while those of SW480 and LoVo cells were significantly lower (Figure 2B). For further cellular experiments, two different siRNAs against ATGL (siATGL‐1 and siATGL‐2) were transfected into HCT116 and SW620 cells, and ATGL‐overexpressing lentivirus was transduced into SW480 and LoVo cells. qRT‐PCR and Western blot were used to detect transfection efficiency (Figure 2C‐F).

### 3.3 ATGL promotes CRC cell proliferation

**FIGURE 3:**
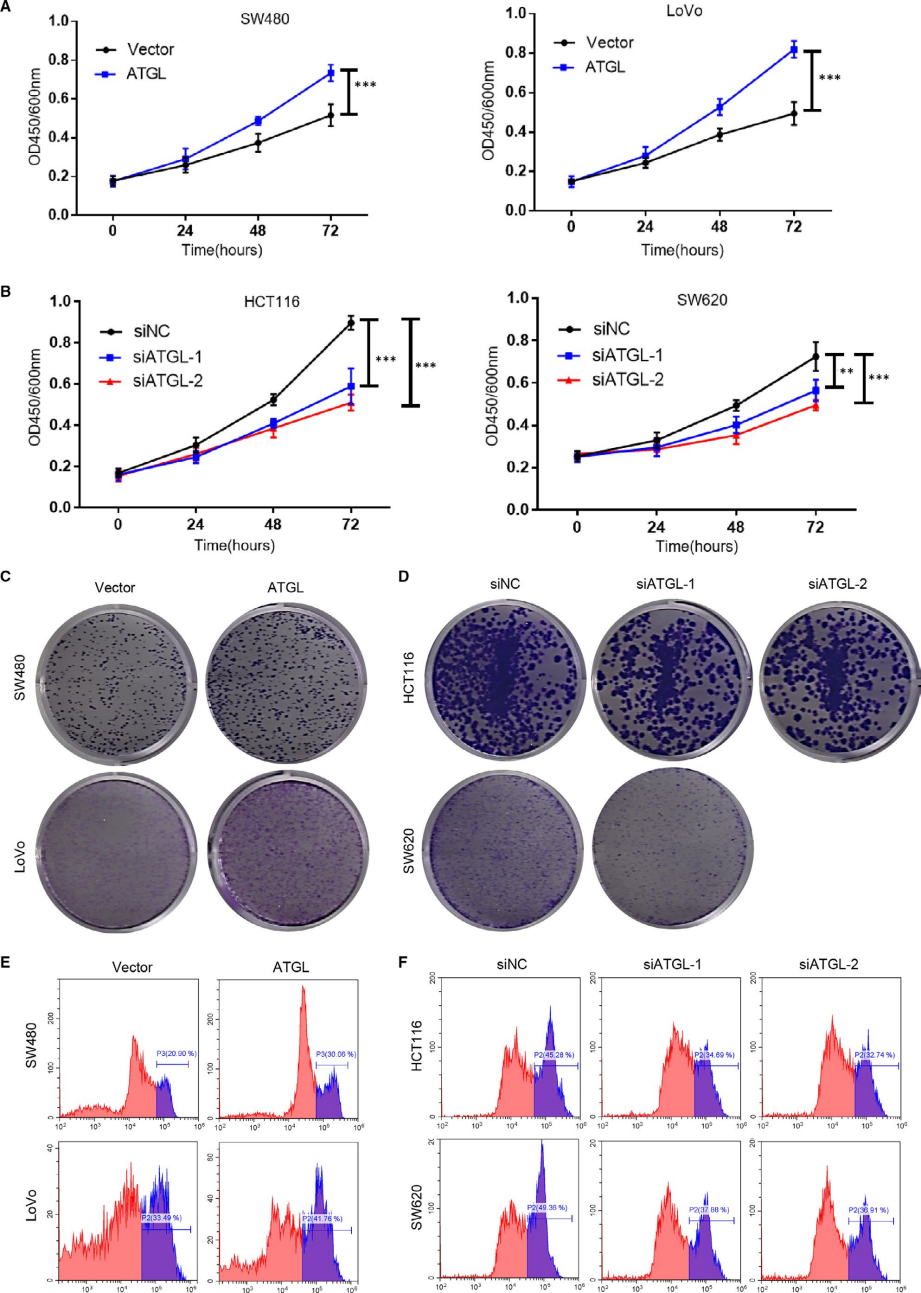
ATGL promotes CRC cell proliferation. (A) CCK‐8 detection for cell proliferation in SW480 and LoVo cells transduced with ATGL viruses and vector control. (B) CCK‐8 detection for cell proliferation in HCT116 and SW620 cells transfected with ATGL siRNA and siNC; **P < 0.01, ***P < 0.001. (C, D) Representative images of colony formation in indicated ATGL‐transduced, ATGL‐silenced or vector control cells. (E, F) EdU staining assay in indicated cells. Data were represented as the mean ± SD of three independent experiments

To reveal the effect of altered ATGL expression on CRC cell proliferation, CRC cells with ATGL overexpression and knockdown were used for CCK‐8, colony formation and EdU staining assays. CCK‐8 results indicated that overexpression of ATGL significantly enhanced the cell viability of SW480 and LoVo cells, while knockdown of ATGL expression strongly decreased the cell viability of HCT116 and SW620 cells (Figure 3A,B). As expected, ATGL‐transduced SW480 and LoVo cells had more colonies than the vector control cells, whereas down‐regulated in ATGL‐silenced HCT116 and SW620 cells (Figure 3C,D; Figure S1A). Moreover, EdU staining assays suggested that the proliferation ability was significantly promoted in ATGL overexpressed cells while inhibited in ATGL silencing cells (Figure 3E,F; Figure S1B). These data indicated that ATGL enhanced the proliferation of CRC cells.

### 3.4 Knockdown of ATGL promotes apoptosis of CRC cells

**FIGURE 4:**
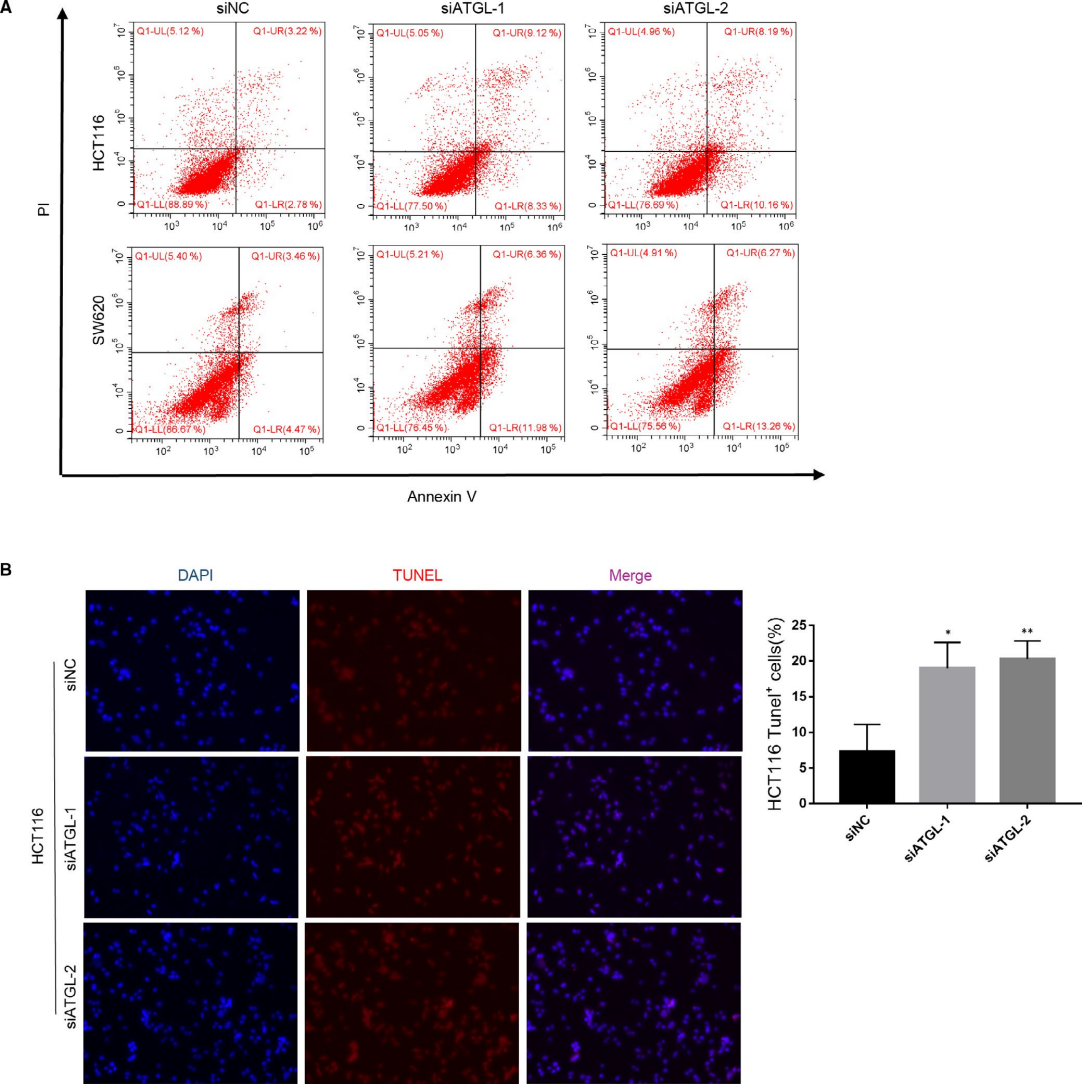
Knockdown of ATGL promotes apoptosis of CRC cells. (A) Detect apoptosis of indicated ATGL‐silenced and control cells by flow cytometry. (B) Representative images of TUNEL staining in HCT116 cells transduced with ATGL siRNA and siNC. All Bars represented the mean ± SD of three independent experiments; *P < 0.05, **P < 0.01

In further experiments, we examined the apoptosis of CRC cells with ATGL knockdown by flow cytometric analysis and TUNEL staining. CRC cells were incubated with PI and Annexin V staining for flow cytometric detection. Our results indicated that the apoptosis rate was apparently elevated after interfering with ATGL (Figure 4A; Figure S2). Furthermore, the results of TUNEL staining suggested that the staining intensity elevated after ATGL knockdown, indicating that knockdown of ATGL promoted CRC cell apoptosis (Figure 4B).

### 3.5 ATGL promotes the lipolytic pathway of CRC cells

**FIGURE 5:**
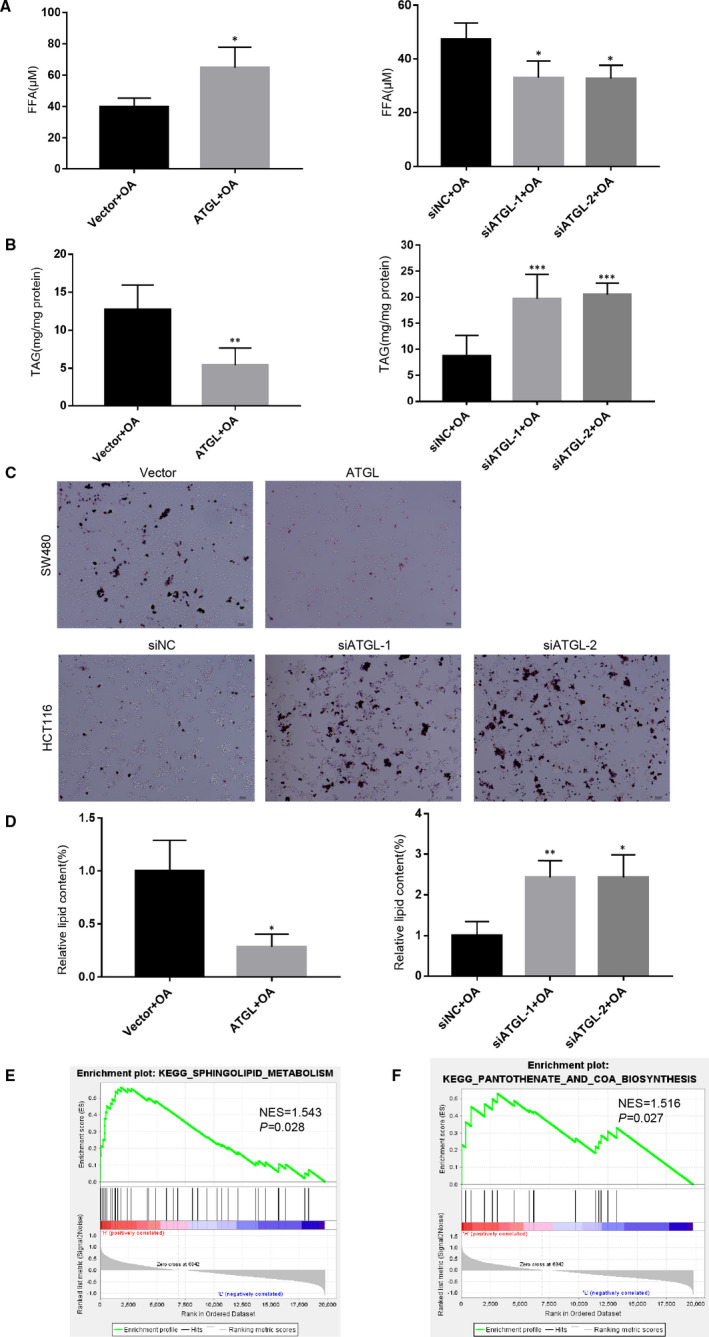
ATGL promotes the lipolytic pathway of CRC cells. (A) Detect the FFA content in indicated SW480 and HCT116 cells culture media after treatment with 400 μM OA for 6 hours; *P < 0.05. (B) Detect the TAG content in indicated SW480 and HCT116 cells after treatment with 400 μM OA for 6 hours; **P < 0.01, ***P < 0.001. (C) Representative images of lipid droplets in indicated SW480 and HCT116 cells after treatment with 400 μM OA for 6 hours and then stained with Oil Red O. (D) Quantification of lipid content are shown. All Bars represented the mean ± SD of three independent experiments; *P < 0.05, **P < 0.01. (E, F) GSEA in the TCGA CRC dataset showed that ATGL were positively correlated with sphingolipid metabolism (E) and CoA biosynthesis (F)

In view of recent reports of ATGL as a crucial regulator of lipid homeostasis, we attempted to explore the role of ATGL lipolytic metabolism in CRC. Firstly, we collected the culture medium of CRC cells after treatment with OA to determine FFA levels. As expected, ATGL‐transduced SW480 cells demonstrated significantly higher FFA and lower TAG content than the control cells, while ATGL‐silenced HCT116 cells showed lower FFA and higher TAG content (Figure 5A,B). Similarly, Oil Red O staining indicated that ATGL‐transduced SW480 cells had evidently fewer intracytoplasmic lipid droplets than the control cells, and the ATGL‐silenced HCT116 cells showed the opposite effect (Figure 5C,D).

To further demonstrate the molecular mechanisms of ATGL in lipid metabolic pathways in CRC, we analysed genes related to ATGL expression in the TCGA CRC dataset. Gene set enrichment analysis (GSEA) indicated that ATGL was positively correlated with sphingolipid metabolism (Figure 5E) and CoA biosynthesis (Figure 5F). The heat map demonstrated that ATGL was positively correlated with ACER2, SMPD1, SPHK2 and other genes in cholesterol metabolism, while ATGL was positively correlated with ENPP1 and other genes in CoA biosynthesis (Figure S3A,B). Bioinformatic analysis results were further verified by qRT‐PCR, and we found that the expression of *ACER2* was most obviously changed in these cells (Figure S3C,D). Collectively, these results suggested that ATGL‐mediated lipolysis releases FFA for cholesterol metabolism and CoA biosynthesis.

### 3.6 ATGL promotes the growth of CRC cells in vivo

**FIGURE 6:**
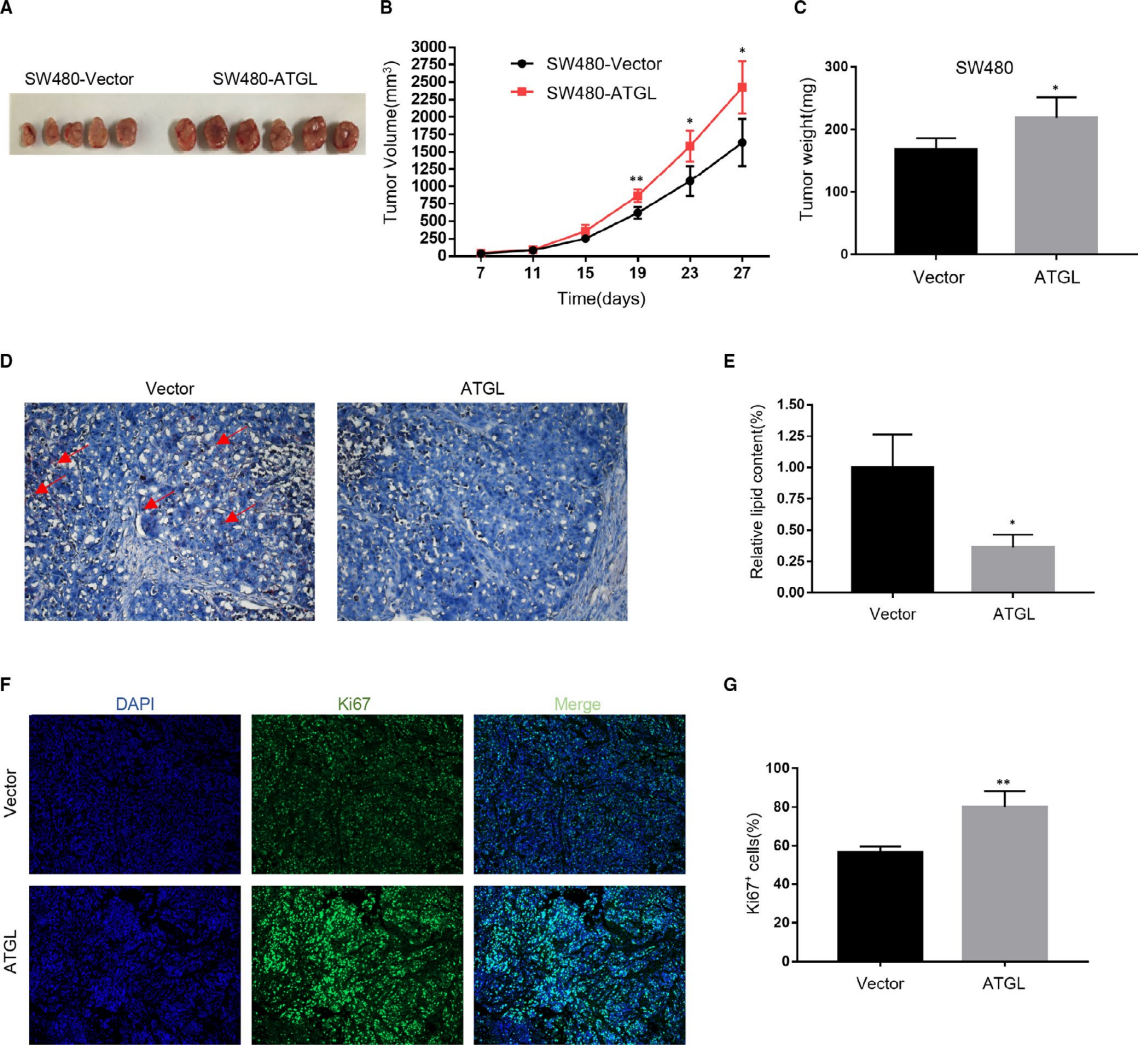
ATGL promotes the growth of CRC cells in vivo. (A‐C) The representative morphology (A), tumour growth rate (B) and tumour weight (C) were presented as a result of SW480‐Vector and SW480‐ATGL Xenograft Models; *P < 0.05, **P < 0.01. (D) Representative lipid droplets images in Xenograft Models tumours. (E) Statistical analysis of lipid droplets; *P < 0.05. (F) Representative Ki67 staining images in Xenograft Models tumours. (G) Statistical analysis of Ki67 staining. Data were expressed as mean ± SD; **P < 0.01

Animal research was performed further to confirm the role of ATGL carcinogenesis in vivo. The tumours generated by ATGL‐transfected SW480 cells had a larger size than those generated by control cells (Figure 6A‐C). Noticeably, Oil Red O staining indicated that ATGL decreased lipid droplets accumulation in SW480 Xenograft Models, which was consistent with the results of in vitro experiments (Figure 6D,E). Moreover, Ki67 staining of the SW480 Xenograft Models tumours showed that ATGL promoted CRC cell proliferation (Figure 6F,G). Our findings revealed that ATGL accelerated CRC cells growth in vivo.

## 4 DISCUSSION

Abnormal lipid metabolism in CRC has been reported to be closely associated with the biological processes of tumorigenesis and development. Lipid mobilization or lipolysis means that one TG molecule is hydrolysed successively by ATGL, HSL and MAGL to release three free FFA molecules and one glycerol molecule.^9^, ^10^ Dysregulated lipid metabolism underlies CRC pathogenesis, facilitating substantial enhancement in FFA content and cancer cell survival. Remarkably, ATGL is the initial enzyme involved in the process of degrades fat into monoglycerides and starts fat mobilization.^9^ However, the relationship between CRC and ATGL remains unclear.

To clarify the role of ATGL in CRC, the expression of ATGL in CRC tissue was examined by IHC. Our results showed that ATGL expression was remarkable elevated in CRC and negatively associated with overall survival (Figure 1). Further, overexpression of ATGL apparently enhanced the proliferation of CRC cell in vitro, while down‐regulation of ATGL was the opposite (Figures 3 and 4). Only recent research has emphasized the regulatory role of ATGL in cancer. While the basic role of ATGL in catabolism has been widely studied over the past decade. However, the limited available data about the functions and mechanisms by which ATGL may affect cancer progression remained elusive and controversial.^17^

Most in vitro studies have proposed that the carcinogenic properties of ATGL are consistent with our results. ATGL knockdown reduced the proliferation and invasiveness of non‐small cell lung carcinoma cell lines.^18^ Recent researches indicated that ATGL promoted the proliferation of HCC cell, while it does not affect migration ability.^15^ Additionally, the up‐regulation of ATGL expression in pancreatic ductal adenocarcinoma was related to the adipocytes enriched tumour microenvironment, which contributes to the invasiveness of high‐grade tumours.^19^ Similarly, breast cancer with increased ATGL expression was characterized by higher adiposity and clinical grades.^20^

Strikingly, recent in vivo studies have revealed the anti‐tumour effects of ATGL. ATGL‐KO mice spontaneously developed pulmonary neoplasia, and specific knockdown of ATGL and HSL in brown adipose tissue can induce liposarcoma.^21^ But our results indicated that ATGL promoted the growth of CRC cells in vivo (Figure 6). And ATGL expression in lung adenocarcinoma was significantly lower than in normal epithelium.^22^ Similarly, the level of ATGL was also decreased in malignant smooth muscle tumours compared with normal controls.^22^ However, our results indicated that both ATGL mRNA and protein levels of CRC cell lines were remarkably elevated than that of immortalized CCD841 human intestinal epithelial cell line (Figure 2A,B). While the role of ATGL in cancer remains controversial due to inconsistent evidence, our results demonstrated the oncogenic role of ATGL in CRC.

The tumour microenvironment as an important regulator in carcinogenesis has been extensively recognized. Increasingly researches focus on the tumour environment to reveal the complicated mechanisms of tumorigenesis and progression.^23^, ^24^, ^25^ Because CRC cells grow in a lipid‐surrounded microenvironment, understanding the link between CRC tumour and lipid can provide novel critical therapeutic target.^26^ As mentioned above, ATGL is a critical lipase in TG hydrolysis, accounting for about 95% of the hydrolysis activity.^7^ Our current results show that CRC cells with high expression of ATGL have strong fat degradation ability, and FFA released by lipolysis can be transferred to other tumour cells through paracrine or autocrine for further use (Figure 5A‐C).

FFA is involved in cancer progression through multiple mechanisms.^11^ For example, it provides energy through β oxidation, and it is part of membrane phospholipids. FFA can not only promotes the biosynthesis of tumour lipid signalling molecules, but also stimulates ligand transcription factor, such as peroxisome proliferator‐activated receptors (PPARs).^27^, ^28^ Recent studies have reported that ATGL promoted the transport of FFA released by fat degradation into the nucleus, activated PPARs, and then activated downstream oncogenes in HCC.^29^, ^30^ However, through the analysis of TCGA data, we did not find that ATGL could promote the PPAR signalling pathway in CRC. GSEA data suggested that ATGL was positively correlated with sphingolipid metabolism and the CoA synthesis pathway, which indicated that ATGL accelerated CRC cell proliferation by up‐regulating the biosynthesis of tumour lipid signalling molecules (Figure 5D,E). Combining bioinformatic analysis and qRT‐PCR results, we found that the expression of *ACER2* was most obviously changed in these cells (Figure S3A,B). Previous study has shown that *ACER2* was the vital gene in the biosynthesis of sphingosine‐1‐phosphate (S1P), which was one of the most vital final products of the sphingolipid metabolism.^31^ It is worthy to further explore whether S1P or other specific lipids responsible for the oncogenic role of ATGL in our further study, which can unravel the precise mechanistic function of ATGL.

Although our current study complements previous studies, the mechanism of ATGL promoting CRC progression has not been fully elucidated. In the future work, some reported mechanisms of ATGL participation can be used as references. In most immunocytes, ATGL promotes the production of cytokine IL‐6 to enhance the pro‐inflammatory response and chemotaxis.^32^, ^33^ Interestingly, clinicopathological characteristics showed a negative correlation between ATGL and CD8 positive T‐cell rates (Table. 1). The occurrence and development of CRC are closely associated with inflammation.^34^ It is worth getting more convincing data to explore the relationship between ATGL and inflammation in CRC. Besides, a recent study showed that ATGL stimulated autophagy and lipophagocytosis in the liver through SIRT1 signalling.^35^ Actually, the ATGL protein sequence contains the LC3 interaction region, which is a motif that mediates the connection between LC3‐coated autophagosomes and autophagy receptor.^36^ The connection between ATGL and LC3 contributes to the degradation of lipids more effectively through the synergy of lipolysis and lipophagy. Hence, the role of ATGL induced autophagy in CRC deserves further exploration.

In summary, our research suggests that ATGL is highly correlated with the progression of CRC via enhancing the lipolytic pathway. These results indicate ATGL is a promising prognostic marker and therapeutic target for CRC.

## CONFLICT OF INTERESTS

The authors declare no conflict of interest.

## AUTHOR CONTRIBUTIONS

**Haofan Yin:** Conceptualization (lead); Data curation (lead); Formal analysis (lead). **Wentao Li:** Conceptualization (equal); Data curation (equal); Formal analysis (equal); Funding acquisition (equal). **Laiming Mo:** Investigation (equal); Project administration (equal). **Shaotuan Deng:** Methodology (equal). **Weijia Lin:** Methodology (equal). **Caiqi Ma:** Funding acquisition (equal). **Zhaofan Luo:** Funding acquisition (equal); Supervision (lead); Validation (lead). **Chuanghua Luo:** Resources (lead); Software (lead). **Honghai Hong:** Funding acquisition (equal); Visualization (lead); Writing‐original draft (lead); Writing‐review & editing (lead).

## Supporting information

Figure S1

Figure S2

Figure S3

## Data Availability

All data generated or analysed during this research are included in this published article.
