# Supplementary figures and images for: Adipose triglyceride lipase promotes the proliferation of colorectal cancer cells via enhancing the lipolytic pathway
Source: J Cell Mol Med. 2021 Feb 23;25(8):3963–75. doi: 10.1111/jcmm.16349 (PMC8051714; doi:10.1111/jcmm.16349)

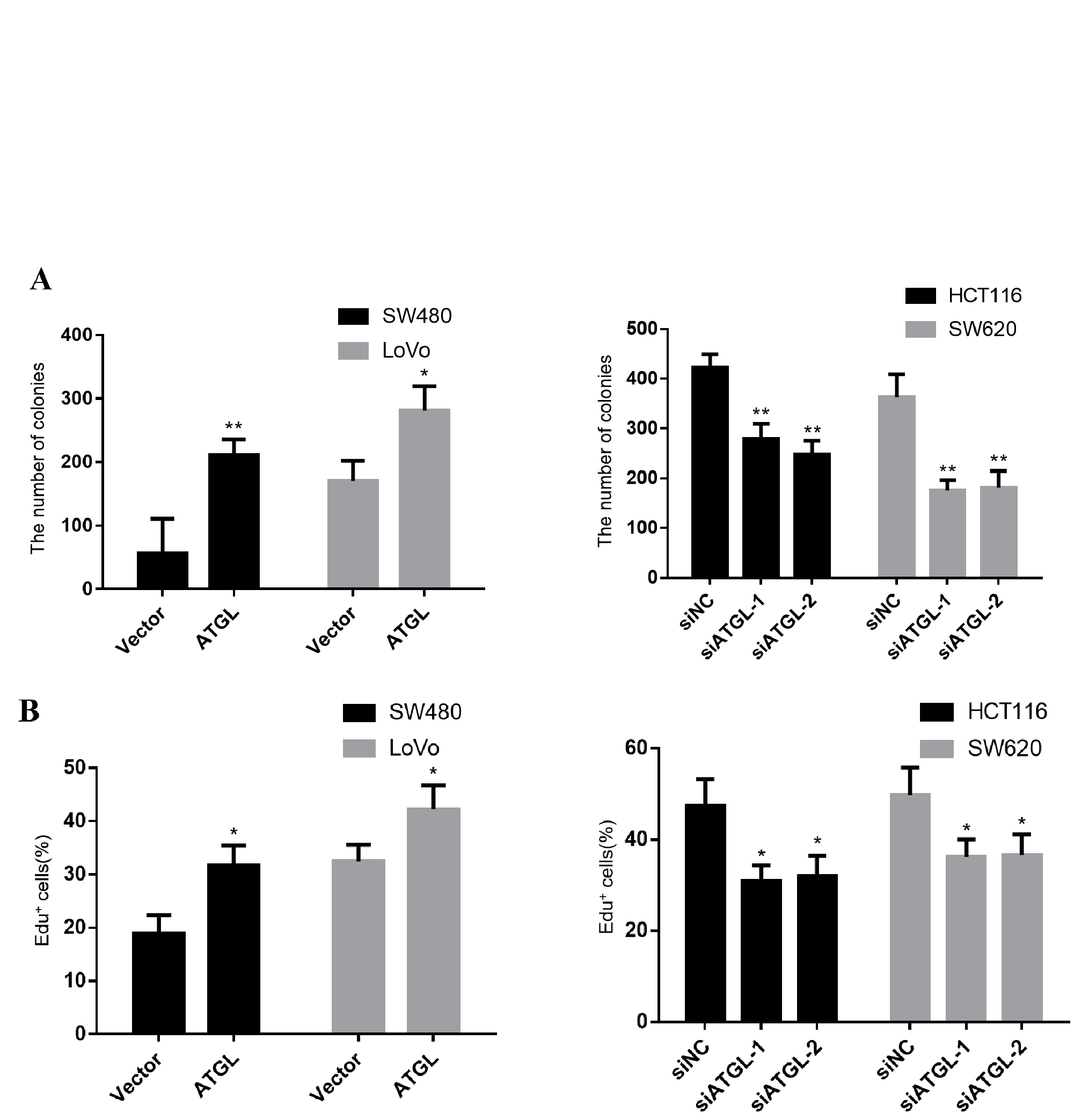

Supplement: Supplementary file 1 — Figure S1 [file JCMM-25-3963-s001.tif]

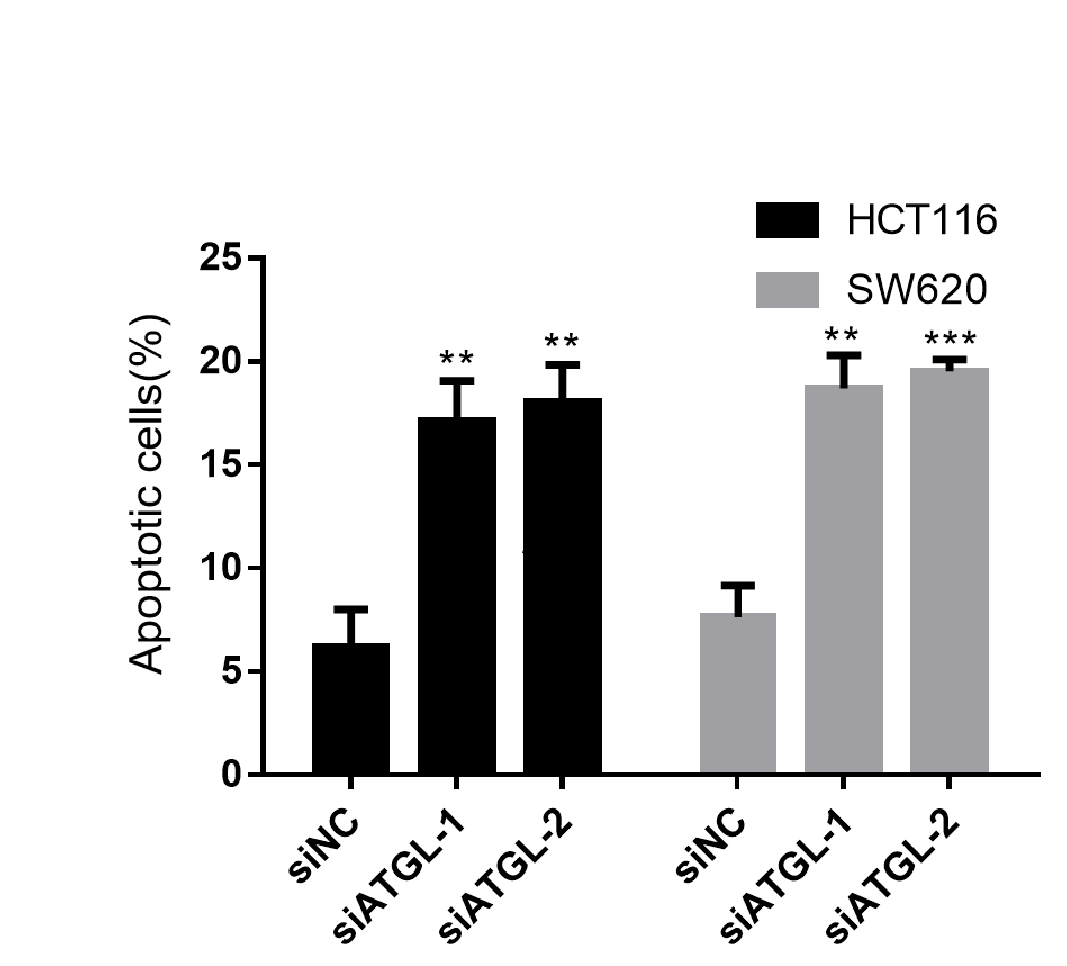

Supplement: Supplementary file 2 — Figure S2 [file JCMM-25-3963-s003.tif]

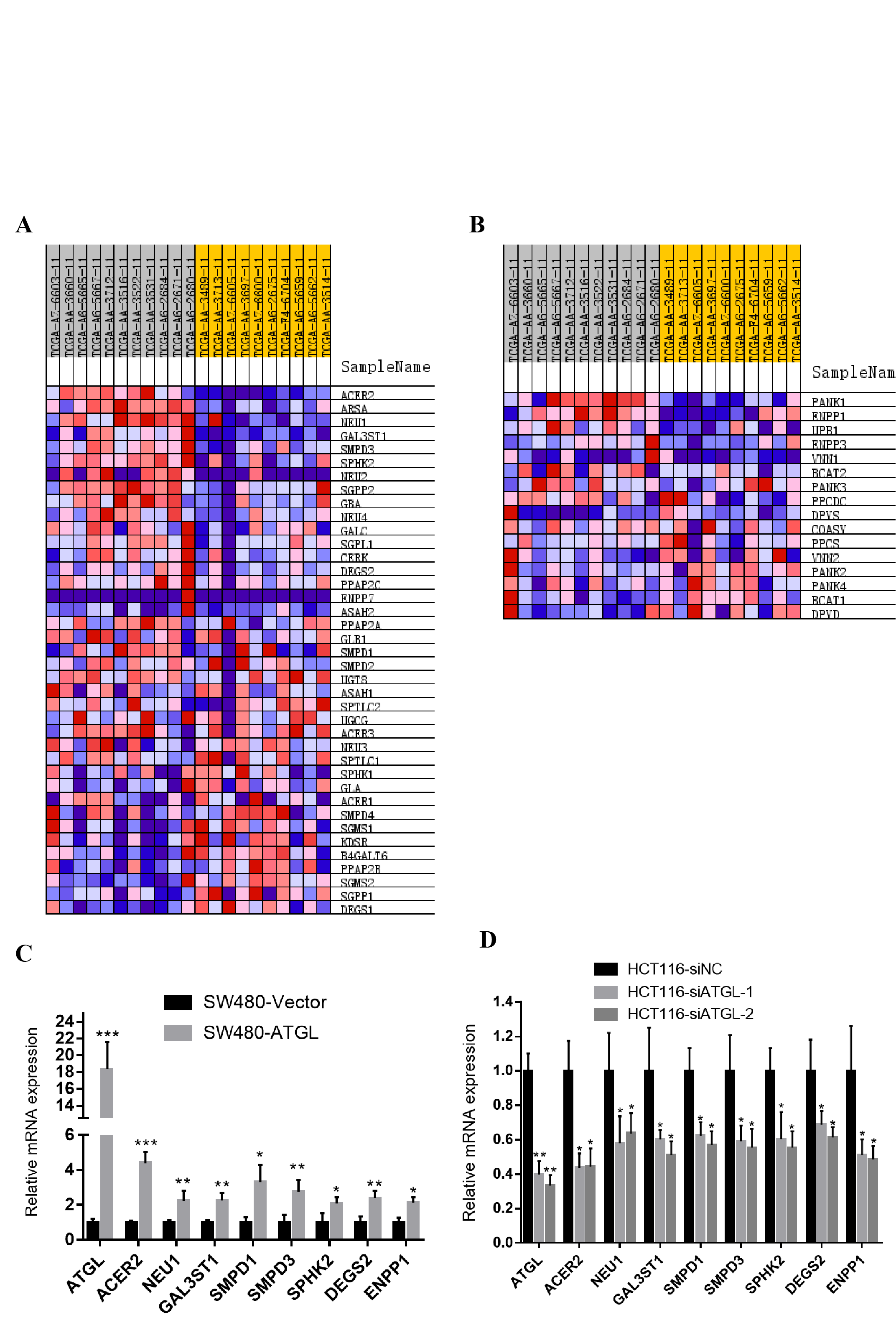

Supplement: Supplementary file 3 — Figure S3 [file JCMM-25-3963-s002.tif]
